# Ecological Effects of Roads on the Plant Diversity of Coastal Wetland in the Yellow River Delta

**DOI:** 10.1155/2014/952051

**Published:** 2014-03-31

**Authors:** Yunzhao Li, Junbao Yu, Kai Ning, Siyao Du, Guangxuan Han, Fanzhu Qu, Guangmei Wang, Yuqin Fu, Chao Zhan

**Affiliations:** ^1^Key Laboratory of Coastal Zone Environmental Processes and Ecological Remediation, Yantai Institute of Coastal Zone Research (YIC), Chinese Academy of Sciences (CAS) and Shandong Provincial Key Laboratory of Coastal Zone Environmental Processes, YICCAS, Yantai, Shandong 264003, China; ^2^University of Chinese Academy of Sciences, Beijing 100049, China; ^3^College of Environmental Science and Engineering, Ocean University of China, Qingdao, Shandong 266100, China

## Abstract

The 26 sample sites in 7 study plots adjacent to asphalt road and earth road in coastal wetland in the Yellow River Delta were selected to quantify plant diversity using quadrat sampling method in plant bloom phase of July and August 2012. The indice of *β*_*T*_ and Jaccard's coefficient were applied to evaluate the species diversity. The results showed that the plant diversities and alien plants were high in the range of 0–20 m to the road verge. There were more exotics and halophytes in plots of asphalt roadside than that of earth roadside. However, proportion of halophytes in habitats of asphalt roadsides was lower than that of earth roadside. By comparing *β*-diversity, there were more common species in the asphalt roadsides than that in the earth roadsides. The similarity of plant communities in studied plots of asphalt roadsides and earth roadsides increased with increasing the distance to road verge. The effect range of roads for plant diversity in study region was about 20 m to road verge. Our results indicate that the construction and maintenance of roads in wetland could increase the plant species diversities of communities and risk of alien species invasion.

## 1. Introduction

Roads are common artificial infrastructures, but high density roads rarely appear in wetlands. However, the study interests of road ecology in the wetland are growing [1–3]. Construction and maintenance of roads have modified the natural wetland landscape and might result in many ecological effects (or ecological risks) [4–6]. Generally, there are six primary ecological effects of roads, that is, (1) habitat loss [7], (2) disturbance [8, 9], (3) corridor [10, 11], (4) mortality [12], (5) barrier [13], and (6) behavior modification [14–16]. The ecological effects of roads can be divided into effects in construction period and short term effects and long term effects in operation period [17, 18]. Therefore, it is difficult to evaluate accurately ecological effects of road on ecosystem because of comprehensive results [19–22]. The roads construction and existence have shown deleterious effects on a variety of ecosystems [1, 23, 24] and often noted by ecologists for their far-reaching negative consequences to ecosystem structures and flows [10, 22, 24–27]. Meanwhile, some road effects are beneficial to ecosystems though they are hard to confirm [22]. For example, ecologists have found that the positive feedbacks, for example, regional climate change, and the amount of forest fragmentation and deforestation directly related to the construction of roads [28, 29]. Actually, it is true that roads and roads edges provide resources for some species, particularly small mammals and insects [7, 17, 24]. In some cases, roads were found to have been acted as essential corridors for survival, movement, and propagation [7, 24, 30]. The wetland vegetation, one of the three most important elements of wetlands [31, 32], is greatly affected by the disturbance of road. The previous studies in the YRD reported that the high species richness and biodiversity were observed adjacent to the road verge [33]. Besides the distance from the road verge, the width, noise, vehicle traffic levels of road, and highway density also could influence the plant community anyway [4, 24, 34]. In addition, the natural wetland landscape and the water-salt migration in coastal wetland could be changed by road, which might result in changes of the environment of vegetation growth [23, 35, 36]. Previous studies reported that there was heavy metals accumulation in road verge soils, which might influence plants adjacent to road indirectly [37–40]. It was also believed that construction and maintenance of roads might alter nutrient levels, bulk density, and moisture of soils and led to a high soil concentration of nitrogen at roadside verges [41, 42]. Roads altered flows of materials in the landscape and changed levels of available resources, such as water, light, and nutrients of ecosystems [24], and thus affected the plant communities. Therefore, it could not be affirmed that the road effects are positive or not briefly before evaluating the road effects objectively as much as possible. To implement this objective, the YRD wetland was selected as a suitable place to evaluate the effects of road on vegetation distribution in coastal wetlands because the high density of roads was constructed for oil exploitation.

There are several types of roads, for example, asphalt road, cement road, and earth road for oil exploitation in the YRD wetland which is a short-formed and protogenous ecosystem with various kinds of wetland plants [43], resulting in dividing the wetland into patches. Our assumption is that the wetland plants might be impacted by different kinds of roads, resulting in differences of community composition and biodiversity appearing in verges of the different types of road. With this assumption, the wetland plant communities beside the road verges of asphalt road and earth road which were representative road types in the study region were surveyed using quadrat sampling method in plant bloom phase. The objectives of this study are to reveal (1) the composition changes of plant communities in the habitats adjacent to the asphalt roads and earth roads and (2) how the plant biodiversity is affected by roads.

## 2. Materials and Methods

### 2.1. Description of the Study Area

**Figure 1:**
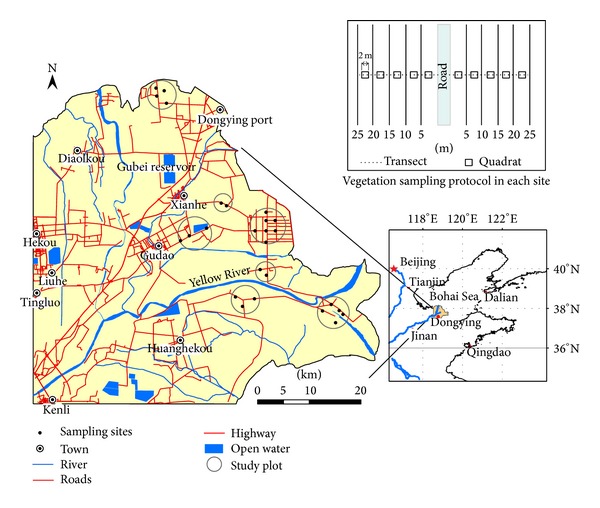
Location of the study area and sampling sites in the Yellow River Delta.

The study area is located in the YRD, eastern China (Figure 1). The regional climate is a temperate semihumid continental monsoon climate and the average annual precipitation in the study area is 530–630 mm, of which 70% is in the summer [44, 45]. Evaporation is strong and the ratio of evaporation to precipitation is about 3 : 1 [46]. The YRD is a flat floodplain with a plain slope of 0.0001 and an area less than 10 m in elevation [47]. Swamp and salt marsh are widespread in the study area and the predominant natural wetland plants are* Phragmites australis, Tamarix chinensis, *and* Suaeda salsa*. The oil exploitation is the major human activity in the natural wetland region where there are few settlements. There was about 2035.5 km of asphalt road built for oil exploitation from 1963 to 2002 in the YRD.

### 2.2. Sampling Sites and Methods

The 26 sample sites in 7 study plots adjacent to asphalt road and earth road in coastal wetlands were selected to quantify plant communities using quadrat sampling method in plant bloom phase of July and August 2012 (Figure 1). The selected study plots were plain and far from human settlements and seashore as well as the Yellow River to weaken the impacts from slope, irrelevant human activities, the sea and river. By referring to the species-area curve established by Zeng et al. [33], the 2 m × 2 m quadrats were adopted for sampling. Two transects with opposite directions perpendicular to a selected road were made in each site. The five quadrats were arranged at 0–5 m, 5–10 m, 10–15 m, 15–20 m, and 20–25 m to the road verge in each transect (Figure 1). The presence, name, coverage, number, and dominance of all vascular plants were surveyed and recorded at each quadrat.

### 2.3. Data Analysis

To reveal the composition of wetland plant communities in the road verges, the proportion of halophytes and exotics in all plants was calculated. The occurrence frequencies of all recorded plants were compared to determine the most common species.

*β*-diversity, which is the variation in species composition among localities [48], was used to detect changes of communities in studied plots with distance to road verges. The indice of *β*_*T*_ which was described by Wilson and Shmida [49] was adopted to implement in this study:

(1) $$\begin{array}{c} \beta_{T}\;=\frac{\left[g\left(H\right)+l\left(H\right)\right]}{2\alpha }, \end{array}$$

where *g*(*H*) and *l*(*H*) are the numbers of species gained and lost, respectively, along a (habitat) gradient and *α* is the average number of species found within the community samples.

The Jaccard's coefficient (*J*) which may be expressed in several ways [50] was used to compare the similarity of plants composition among localities:

(2) $$\begin{array}{c} J=\frac{c}{a+b-c}, \end{array}$$

where *c* is the number of species common to both sites, *a* is the number of species in the first plot (with high species), and *b* is the number of species in the second plot (with low species).

In addition, independent-sample Student's *t*-test was used to evaluate the effects of regional disparity on number of species applying the software of SPSS 18.0 [51].

## 3. Results and Discussion

### 3.1. Comparison of Plant Species in the Asphalt Roadside and Earth Roadside

A total of 48 plant species with an incidence of 10–90% for eighteen species were found within 25 m to the roadsides during investigation (Table 1). The observed species belonged to 22 families. The best-represented families were Gramineae (8), Compositae (7), Chenopodiaceae (5), and Leguminosae (4). The most common species were* Phragmites australis *(Cav.) Trin. ex Steud.*, Suaeda salsa *(L.) Pall.*, Tamarix chinensis *Lour.*, Sonchus oleraceus *L.*, Artemisia argyi *Levl. et Van., and* Imperata cylindrica *(L.) Beauv.Eight species (17%) were identified as exotics and 36 (75%) as halophytes. The most abundant life form was herb, of which 40 species (83%) were observed. The total observed plant species in this study was about half of previous identified results (100 species) in the same region [33, 52], because of the attributions of the different sampling sites location, roadside conditions, and anthropogenic impacts. The sampling sites in the previous study mainly located in both sides of truck road in the central part of the YRD where the conditions of soil and freshwater for plant were much better than those in present plots. In addition, some special plants were purposefully planted in truck road verges to maintain the roadbed and the high vehicle flow increased the spread of exotics, resulting in higher alien plants in previous study [33, 52] than this study. Agreeing with previous studies [33, 52, 53], the herbaceous plants with high proportion of halophytes were high and ligneous plants were few in the communities of roadsides in the YRD (Table 1).

**Figure 2:**
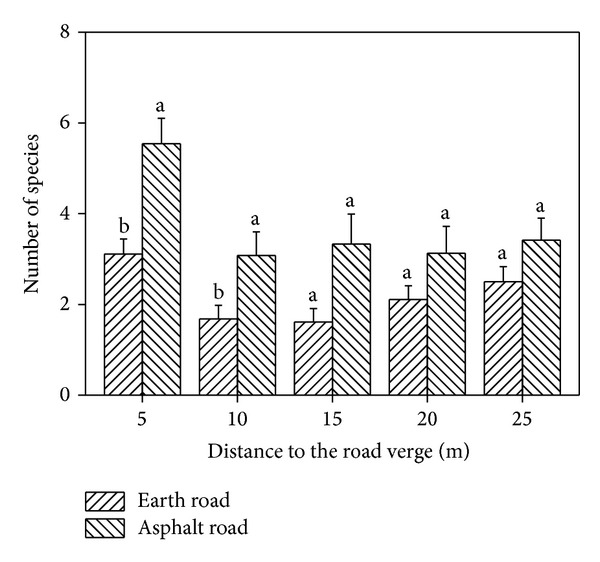
Number of species in plots of asphalt roadsides and earth roadsides. Letters above error bar of each column indicate significant difference at the *P* < 0.05 level for Student's *t*-test.

**Figure 3:**
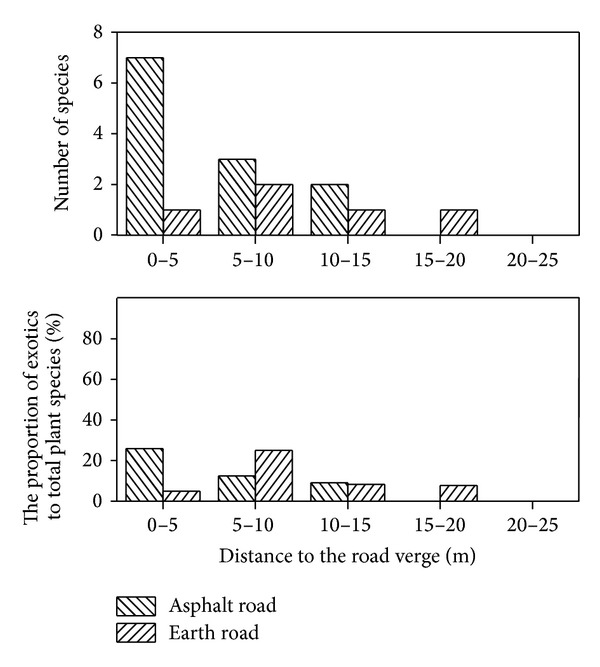
Number and proportion of alien plant species in plots of asphalt roadsides and earth roadsides.

**Figure 4:**
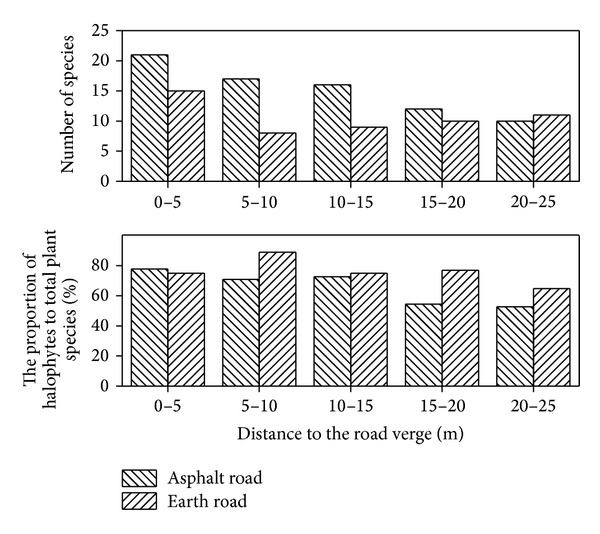
Number and proportion of halophytes in plots of asphalt roadsides and earth roadsides.

The mean value of species in the asphalt roadside was larger than that in the earth roadside regardless of distance from road verges (Figure 2). The differences of number of species were significant within 0–10 m from road edge between the earth road and the asphalt road (Figure 2). Generally, the number of exotics decreased with distance increase to the road edge and those in asphalt roadside were greater than that in earth roadside regardless of distance from road verge (Figure 3). 24 (80%) and 30 (77%) halophytes were found in the earth road verge and the asphalt road verge, respectively (Table 1). The number of halophytes in the asphalt roadside was greater than that in the earth roadside. However, the proportion of halophytes in all plant species in the asphalt roadside was smaller than that in the earth roadside (Figure 4). The differences of environment and the anthropogenic activities of the asphalt roadside and earth roadside were mainly responsible for the plant species difference. The truck numbers and human being activities in the asphalt road are much more than the earth road. Based on the survey results, the vehicle traffic flow in asphalt roads was about 5–8 times higher than that in earth roads, resulting in both the native and the alien plants species in the plots of asphalt roadside being more abundant than that of the earth roadside within a certain distance from sampling site to road (Figures 2 and 3). Furthermore, in order to protect the road and avoid soil-water loss, the vegetation beside the asphalt road was removed first, and then different species of trees and bushes were planted. The removal of habitats and vegetation were soon replaced by new settlers, resulting in more edges in landscape and more niches in communities in the asphalt roadside [4, 18, 54].

### 3.2. Effects of Roads on Plant Communities

Roads increased the contacts between plant communities [24] and the anthropogenic activities near roadsides had great impacts on species [3, 4]. In this study, there were 39 species, of which thirty-two species (82%) were categorized as herbs (Table 1) and were found in 24 sampling transects in the asphalt roadsides, while there were 30 species of which 24 species (80%) were grassland species and were observed in 28 sampling transects in the earth roadsides (Table 1). Additionally, three and seven alien plants were found near the earth roadside and the asphalt road verge, respectively (Table 1). The exotics accounted for no more than 30% of total plants in both asphalt roadside and earth roadside (Figure 3). With the distance to the road edge being increased, the numbers of both saline plants and their proportion in all plants decreased (Figure 4). The results were similar to some previous study results [33, 41, 52]. The road was regarded as dispersal corridor and conduit for vegetation [24, 30, 55–57]. It is believed that roads promoted the dispersal of plant propagules along roadsides [33]. Because of the easy movement of wind, water runoff, and animals by roads, the numerous seeds were carried and deposited along roads by those carriers [4, 58]. Therefore, plants with high dispersal capacities could preferentially occupy their living spaces along roadsides [56, 59, 60]. Furthermore, the dispersal of native and alien plants was affected directly by the vehicle traffic flow and vectors. Roadsides do provide better growth conditions and are good habitat to vegetation [61, 62]. Construction and maintenance of road altered the physical and chemical environment of plant communities [4, 5]. The alterations of light conditions, soil nutrients, and water availability were remunerative for plant communities, thus increasing the survival opportunities for most plants [61, 62]. Additionally, the elevation of most roadbeds was 1-2 meters higher than that of around wetlands in the YRD, which was a great benefit in salt reducing. The areas of roadsides have become refuges to many nonhalophytes. As a result, both native and alien plant species in roadsides were more abundant than wetland far away from road verge and the proportion of halophytes in the road verges was much lower than in wetland habitats (Table 1 and Figure 4).

### 3.3. Diversity of Plant Communities in Roadside

**Figure 5:**
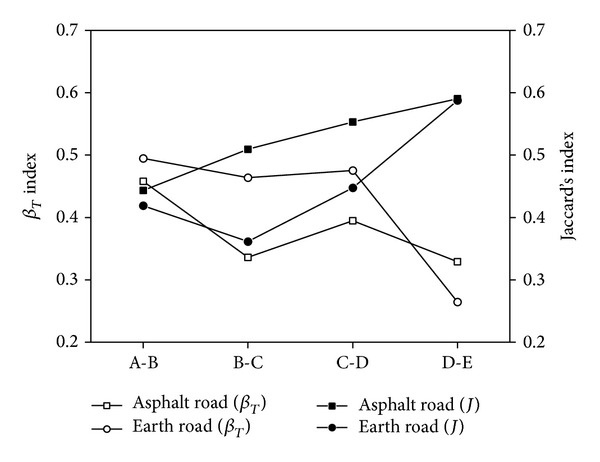
Characteristic of *β*_*T*_ index and Jaccard's coefficient in plots of the asphalt roadsides and earth roadsides. A: plant communities in plots of 0–5 m to road verge; B: plant communities in plots of 5–10 m to road verge; C: plant communities in plots of 10–15 m to road verge; D: plant communities in plots of 15–20 m to road verge; E: plant communities in plots of 20–25 m to road verge.

The variation of *β*_*T*_ index showed different patterns between asphalt roadside and earth roadside (Figure 5 and Table 2). The *β*_*T*_ index of the plant communities decreased gradually with distance to road verge in the spots adjacent to asphalt road. While the variation of *β*_*T*_ showed an inverse “*N*” shape with the distance from plant communities to earth roadside, increased the  *β*_*T*_ index in study sites adjacent to earth roadside was larger than that adjacent to asphalt roadside within 20 meters to road verge (Figure 5). The similarity of communities was completely different between the spots of asphalt roadside and earth roadside (Figure 5). The Jaccard's coefficient gradually increased with increase of the distance from study plot to roadside in the asphalt road verge. However, The Jaccard's coefficient of plant communities in the earth roadside decreased originally and then increased with the distance from study plots to earth roadside increasing (Figure 5). The values of the Jaccard's coefficient in the study plots adjacent to earth roadside were smaller than that adjacent to asphalt roadside within 20 meters from road verge (Figure 5). As soon as the distances of study plots to roadside were more than 20 m, the values of the Jaccard's coefficient in the plots of earth roadside were approximate to that in the asphalt roadside (Figure 5). Our results indicated that both *β*_*T*_ index and Jaccard's coefficient, which provide different but equally illuminating views of biodiversity [63, 64], were adopted in this study. The *β*_*T*_ of plant communities in roadsidetended to decrease, while Jaccard's coefficient increased, with the distance increasing from sampling plots to road verge in asphalt roadsides (Figure 5). Some previous studies indicated that construction and maintenance of roads did a lot of negative effects to plant communities [24, 65, 66] because most communities were destroyed during construction of roads, leaving few survival plants. The patterns of *β*_*T*_ and Jaccard's coefficient indicated low ebb in plant communities within 5 to 15 m of earth roadsides (Figure 5). Actually, during investigation and sampling, the bare land and open water were the most common landscape types in 5 to 15 m away from earth in the YRD. While the sampling sites were more than 20 m away from road verges, the plant communities in both asphalt roadsides and earth roadsides indicated little differences.

## 4. Conclusions

The biodiversity of plant communities are deeply affected by roads in the YRD. The high plant species, especially exotics, was observed in the range of 0–20 m to the road verge. There were more plant species and exotics in the asphalt roadsides than that in the earth roadsides. However, the proportion of halophytes in plant communities in the earth roadsides was higher than that in the asphalt roadsides. The analysis results of *β*-diversity showed that there were more common species in the asphalt roadsides than that in the earth roadsides. The similarity of plant communities in studied plots of asphalt roadsides and earth roadsides increased with the increasing distance to road verge. The effected distance of roads on plant diversity was limited within 20 m to road verge. Our results indicate that the construction and maintenance of roads in wetland could increase the plant species diversities of communities and risk of alien species invasion.

## Figures and Tables

**Table 1:** List  of  plant  species  distributed  in  roadsides  (range  of  25 m)  of  the  Yellow  River  Delta during  our  investigation.

Species	Family	Plots of earth roadside	Plots of asphalt roadside	Halophyte	Exotics	Life form
*Amaranthus retroflexus *	Amaranthaceae		Y			Herb
*Amaranthus viridis *	Amaranthaceae	Y	Y	Y	Y	Herb
*Rhus typhina *L.	Anacardiaceae		Y		Y	Arbor
*Apocynum venetum *L.	Apocynaceae	Y	Y	Y		Shrub
*Metaplexisjaponica* (Thunb.) Makino	Asclepiadaceae	Y		Y		Liane
*Cynanchum chinense *R. Br.	Asclepiadaceae	Y	Y	Y		Liane
*Atriplex centralasiatica *Iljin	Chenopodiaceae	Y		Y		Herb
*Chenopodium hybridum *L.	Chenopodiaceae	Y		Y	Y	Herb
*Suaeda salsa* (L.) Pall.	Chenopodiaceae	Y	Y	Y		Herb
*Suaeda glauca* (Bunge) Bunge	Chenopodiaceae	Y	Y	Y		Herb
*Kochiascoparia* (L.) Schrad.	Chenopodiaceae	Y		Y		Herb
*Cirsium maackii *Maxim*. *	Compositae		Y			Herb
*Taraxacum mongolicum *Hand.-Mazz.	Compositae		Y	Y		Herb
*Artemisia argyi *Levl. et Van.	Compositae	Y	Y	Y		Herb
*Scorzonera mongolica *Maxim.	Compositae		Y	Y		Herb
*Artemisia capillaris *	Compositae	Y		Y		Herb
*Sonchus oleraceus *L.	Compositae	Y	Y	Y		Herb
*Dendranthema indicum* (L.) Des Moul.	Compositae	Y	Y	Y		Herb
*Pharbitis nil* (L.) Choisy	Convolvulaceae		Y	Y		Herb
*Convolvulus arvensis *L.	Convolvulaceae		Y		Y	Herb
*Cuscuta chinensis *Lam.	Convolvulaceae	Y	Y	Y	Y	Cancerroot
*Cyperus rotundus* L.	Cyperaceae	Y				Herb
*Carex tristachya *Thunb.	Cyperaceae	Y	Y			Herb
*Ephedra sinica *Stapf	Ephedraceae Dumortier		Y	Y		Herb
*Imperata cylindrica *(L.) Beauv.	Gramineae	Y	Y	Y		Herb
*Triarrhena sacchariflora* (Maxim.) Nakai	Gramineae	Y				Herb
*Setaria viridis* (L.) Beauv.	Gramineae		Y	Y		Herb
*Chloris virgata *Sw.	Gramineae		Y	Y		Herb
*Alopecurus aequalis *Sobol.	Gramineae	Y				Herb
*Phragmites australis* (Cav.) Trin. ex Steud.	Gramineae	Y	Y	Y		Herb
*Aeluropus sinensis* (Debeaux) Tzvel.	Gramineae	Y	Y	Y		Herb
*Calamagrostis epigeios* (L.) Roth	Gramineae		Y	Y		Herb
*Leonurus artemisia* (Laur.) S. Y. Hu	Labiatae		Y	Y		Herb
*Melilotus officinalis *	Leguminosae		Y	Y	Y	Herb
*Robinia pseudoacacia *	Leguminosae		Y			Arbor
*Medicago sativa *L.	Leguminosae	Y	Y	Y		Herb
*Glycine soja *Sieb. et Zucc.	Leguminosae	Y	Y	Y		Herb
*Humulus scandens *	Moraceae	Y	Y	Y		Herb
*Epilobium hirsutum *L.	Onagraceae	Y	Y	Y		Herb
*Limonium bicolor *(Bag.) Kuntze	Plumbaginaceae	Y		Y		Herb
*Polygonum orientale *L.	Polygonaceae		Y	Y		Herb
*Polygonum lapathifolium *L.	Polygonaceae	Y	Y			Herb
*Portulaca oleracea *L.	Portulacaceae		Y	Y		Herb
*Salix matsudana *	Salicaceae	Y	Y			Arbor
*Veronica didyma *Tenore	Scrophulariaceae		Y		Y	Herb
*Datura stramonium *Linn.	Solanaceae		Y	Y	Y	Herb
*Tamarix chinensis *Lour.	Tamaricaceae	Y	Y	Y		Shrub
*Typha orientalis *	Typhaceae	Y	Y	Y		Herb

**Table 2:** Matrix of *β*-diversity indices measured by numerical data from sampling sites.

Distance of sampling sites from road (m)	0–5	5–10	10–15	15–20	20–25
Asphalt roadside					
0–5	×	0.4581	0.5445	0.5751	0.5672
5–10		×	0.3361	0.5408	0.5308
10–15			×	0.3949	0.4494
15–20				×	0.3291
20–25					×
Earth roadside					
0–5	×	0.4946	0.5403	0.5342	0.5242
5–10		×	0.4639	0.6117	0.6082
10–15			×	0.4753	0.4689
15–20				×	0.2362
20–25					×
